# Enhanced Oral Bioavailability, Anti-Tumor Activity and Hepatoprotective Effect of 6-Shogaol Loaded in a Type of Novel Micelles of Polyethylene Glycol and Linoleic Acid Conjugate

**DOI:** 10.3390/pharmaceutics11030107

**Published:** 2019-03-06

**Authors:** Huiyun Zhang, Qilong Wang, Congyong Sun, Yuan Zhu, Qiuxuan Yang, Qiuyu Wei, Jiaxin Chen, Wenwen Deng, Michael Adu-Frimpong, Jiangnan Yu, Ximing Xu

**Affiliations:** Center for Nano Drug/Gene Delivery and Tissue Engineering, Department of Pharmaceutics, School of Pharmacy, Jiangsu University, Zhenjiang 212013, China; zhanghuiyun1111@163.com (H.Z.); wql001wql001@163.com (Q.W.); horizonpacino@sina.cn (C.S.); zhuyuanemail@126.com (Y.Z.); yangqiuxuan1016@126.com (Q.Y.); weiqiuyu0922@163.com (Q.W.); 18352864633@163.com (J.C.); deng4956@yeah.net (W.D.); 5103160103@stmail.ujs.edu.cn (M.A.-F.)

**Keywords:** 6-shogaol, cytotoxic effect, HepG2 cells, micelles, hepatoprotection

## Abstract

6-shogaol is a promising anti-cancer and anti-inflammatory agent. However, the treatment effectiveness of 6-shogaol is limited by poor water solubility, poor oral absorption and rapid metabolism. Herein, 6-shogaol loaded in micelles (SMs) were designed to improve 6-shogaol’s solubility and bioavailability. The micelles of a PEG derivative of linoleic acid (mPEG_2k_-LA) were prepared by the nanoprecipitation method with a particle size of 76.8 nm, and entrapment of 81.6 %. Intriguingly, SMs showed a slower release in phosphate buffer saline (PBS) (pH = 7.4) compared to free 6-shogaol while its oral bioavailability increased by 3.2–fold in vivo. More importantly, the in vitro cytotoxic effect in HepG2 cells of SMs was significantly higher than free 6-shogaol. Furthermore, SMs could significantly improve the tissue distribution of 6-shogaol, especially liver and brain. Finally, SMs showed a better hepatoprotective effect against carbon tetrachloride (CCl4)-induced hepatic injury in vivo than free 6-shogaol. These results suggest that the novel micelles could potentiate the activities of 6-shogaol in cancer treatment and hepatoprotection.

## 1. Introduction

Ginger, the dried rhizome of *Zingiber officinale*, is not only a popular spice and seasoning, but also a traditional Chinese medicine used to treat many diseases such as nausea, diarrhea and cold [1]. Recently, gingerols, the main chemical components of ginger, have been found responsible for pharmacological effects including analgesic [2], antipyretic [3], cardiotonic [4], hypothermia inducing [5], and cancer prevention [6]. 6-shogaol is one of the shogaols obtained from Gingerols via dehydration during processing and storage. 6-shogaol exhibits significant antioxidant, anti-proliferative and anti-inflammation activity [7]. 6-shogaol is reported to induce cell cycle arrest coupled with autophagy and apoptosis in various cancer cells including hepatocellular carcinoma (HCC) cell, human non-small cell lung cancer A549 cells and human colon adenocarcinoma (HT-29) cells [8,9,10]. 6-shogaol has also been previously reported to possess anti-inflammatory effects by downregulating nitric oxide synthase and COX-2 gene expression [11], while reducing the levels of β-glucuronidase and lactate dehydrogenase [12].

The potentiality of 6-shogaol is supported by several lines of evidence as an agent for the prevention and treatment of a variety of cancers including gastrointestinal, melanoma, breast, lung, head and neck [13,14,15,16,17]. Also, various in vitro and in vivo studies showed that 6-shogaol demonstrates more potent anti-inflammatory activity than 6-gingerol and another widely studied phytochemical, curcumin [18]. However, low bioavailability alongside poor solubility of 6-shogaol hinder its clinical application, probably due to poor absorption, hydrophobicity, extreme instability, rapid metabolism, with concomitant elimination [19,20]. Recently, nanotechnology (polymer nanoparticles or micelles, liposomes, inorganic nanoparticles and nano-emulsions) have shown a huge advantage in enhancing the solubility of lipophilic compounds, oral absorption, bioavailability as well as reducing medicinal herb doses and toxicity, thereby improving the target ability and therapeutic effect compared with traditional Chinese herbal preparations [21,22]. Among them, micelles have already received a lot of attention due to their unique peculiarities like a hydrophilic outer shell coupled with a hydrophobic inner core. These qualities impart on micelles’ water dissolvability, which result in the spontaneous production of micelles with the capability of encapsulating and solubilizing poorly aqueous-soluble drugs [23,24]. Comparatively, self-assembled micelles could provide several advantages to drug delivery systems because of their high drug loading capacity, low dose of formulation required and long circulation time [25,26].

Currently, several PEGylated small amphiphilic molecules such as D-α-tocopheryl polyethylene glycol succinate (TPGS), amphiphilic PEG1000-DOX conjugate and PEG-all trans-retinoic acid conjugates are being used as materials for the fabrication of micelles [27,28,29]. Compared with clinical formulation, docetaxel-loaded TPGS2k micelles showed a lower critical micelle concentration (CMC) and achieved a sustained and controlled drug delivery. Furthermore, docetaxel-loaded TPGS2k micelles enhanced the cytotoxicity on cancer cell lines with the side effects of docetaxel being modulated [30]. As a P-glycoprotein (P-gp) inhibitor, TPGS micelles for oral delivery of paclitaxel have been demonstrated to have superior performance in vivo as evidenced by their enhanced absorption and biological availability [31]. Encapsulating 6-shogaol in micelles will therefore gain benefits from the aforementioned advantages that are associated with such a micelle delivery system.

In the present study, PEG derivative of linoleic acid (mPEG_2K_-LA) was firstly employed as a material for forming micelles to encapsulate 6-shogaol and enhance its solubility. The formulated 6-shogaol loaded micelles (SMs) significantly slowed the drug release in stimulated media of gastro-intestinal tract and increased the sensitivity of tumor cells to the prototype drug. The oral bioavailability, tissue biodistribution and hepatoprotective activity were investigated to evaluate the effectiveness of SMs in delivering 6-shogaol, as well as its bioactivity in vivo. Collectively, this study could provide an experimental basis for the further development and application of self-assembly nano drug delivery systems for enhancing the bioactivity of hydrophobic drugs.

## 2. Materials and Methods

### 2.1. Materials

6-shogaol (98% purity, analytical-grade reagent) was purchased from Aladdin Industrial Corporation (Shanghai, China). Curcumin was obtained from J&K Scientific Co. Ltd. (Beijing, China). Ginger extractive (10% gingerol) was purchased from Nanjing Zelang Biological Technology Co. Ltd. (Nanjing, China). mPEG_2K_-LA was synthesized in our laboratory [32]. (3-(4,5-Dimethylthiazol-2-yl)-2,5-diphenyltetrazolium bromide (MTT) and trypsin were provided by the Beyotime Institute of Biotechnology (Jiangsu, China). Diagnostic kits for assaying serum aspartate aminotransferase (AST), alanine aminotransferase (ALT) and the glutathione peroxidase (GSH-Px), total superoxide dismutase (T-SOD) and the levels of malondialdehyde (MDA) were purchased from Nanjing Jian Cheng Bioengineering institute (Nanjing, China). Chromatographically pure methanol and acetonitrile were procured from Sinopharm Chemical Reagent Technology Co. Ltd. (Shanghai, China).

### 2.2. Preparation of 6-Shogaol from Ginger Extractive

Concisely, the ginger extract (150 g) was first extracted with ethyl acetate, and the organic solvent layer was concentrated up to dryness under a rotary evaporator. Then, a silica gel column was used to separate the 6-shogaol from ethyl acetate extract with gradient elution consisting of petroleum ether and ethyl acetate (5:1, 4:1, 3:1, 2:1 and 5:3) with 500 mL of each mixture. The crude samples were purified further using a C18 silica gel column and eluted with different proportions of methanol and water with ratios ranging from 50/50 to 100/0. The obtained purified samples were identified with ESI-MS (Figure S1) and ^1^H-NMR (Figure S2). According to the calibration curve of HPLC, the obtained 6-shogaol had a purity of 96.67%. The chemical structure was provided in Figure S3. Chemical formula: C_17_H_24_O_3_. ESI-MS (m/z): 299.16 [M+Na]^+^. ^1^H-NMR (400 MHz, CDCl_3_, ppm): δ 0.91 (t, 3H, –CH_3_), 1.25–1.38 (m, 4H, –CH_2_), 1.47 (dt, 2H, *J* = 7.3 and 14.6 Hz, –CH_2_–), 2.21 (2H, dd, *J* = 6.3 and 14.0 Hz), 2.87 (4H, tt, *J* = 5.9 and 12.0 Hz, –CH_2_– between Ph ring and ketone), 3.88 (s, 3H, -OMe), 6.11 (1H, dt, *J* = 1.5 and 15.9 Hz, =CH–), 6.68 (1H, dd, *J* = 2.0 and 8.0 Hz, =CH–), 6.72 (1H, d, *J* = 1.8 Hz, ArH), 6.84 (2H, m, ArH).

### 2.3. Solubility of 6-Shogaol

The solubility of 6-shogaol was determined according to our earlier reported method [22]. Simply, 20 mg of 6-shogaol (20 mg) was added to 1 mL of a different dissolution medium, followed by incubation in a water bath shaker for 100 rpm at 37 °C for 3 days. The suspension was then centrifuged at 10,000 rpm for 20 min to remove the insoluble 6-shogaol. Then, the concentration of supernatant was measured with established HPLC method.

### 2.4. Preparation of 6-Shogaol Loaded Micelles (SMs)

Self-assembled micelles were prepared via the nanoprecipitation method as previously reported with slight modifications [33]. Briefly, 6-shogaol (10 mg) and mPEG_2K_-LA (100 mg) were completely dissolved in 200 µL ethanol solution (200 µL). The ethanol solution was added drop-wise to 2 mL water (2 mL) at room temperature, alongside mechanical stirring (~600—800 revolutions per minute (rpm)) while self-assembly of NMs occurred spontaneously. The organic solvent-free SMs were obtained after evaporating the ethanol in the nano-formulation. The SMs were filtered through a 0.22 µm filter membrane, and then lyophilized prior to storage at 4 °C.

### 2.5. HPLC Analysis Method for Measuring 6-Shogaol Concentration

6-shogaol levels in micelle, plasma and tissue samples were measured via an RP-HPLC method. HPLC analysis was carried out using a Shimadzu Scientific instrument equipped with an LC-20AT pump and an SPD-20A UV-Vis detector (Shimadzu, Kyoto, Japan) on a Symmetric C18 column (4.6 mm × 150 mm, 5 µm, Waters, Milford, MA, USA) with column temperature of 30 °C. The flow rate was set at 1.0 mL/min while the detection wavelength was 230 nm. A 70% methanol-water was chosen as the mobile phase for measuring encapsulation efficiency (EE) and in vitro release, while 65% methanol-water was used for the analysis of bioavailability and tissue biodistribution studies. The system suitability test results of the formulation, PK and tissue analysis methods were depicted in Figures S4–S6. The linear regression equation of 6-shogaol in vitro analysis was Y = 81883X − 44299 (n = 3, *R*^2^ = 0.9997), where Y represented the peak area of samples and X represented the sample concentrations of 6-shogaol. The typical equation of the standard curves of the peak area ratio to the concentration, the linear range and regression coefficients of 6-shogaol in plasma and tissue homogenates were summarized in Table S1. The precision (intra-day and inter-day precision) and accuracy (relative recovery) of the analysis method were assessed at 3 levels of QC samples (in vivo) in different matrices (0.35 μg/mL, 1.5 μg/mL and 7.5 μg/mL in rat plasma; 0.15 μg/mL, 0.375 μg/mL and 0.75 μg/mL in heart, spleen, lung, brain, liver, kidney, stomach and intestine), and the results were shown in Table S2. The extraction recovery of 6-shogaol in plasma, heart, liver, spleen, lung, kidney and brain were within the range of 76.23–87.12%, 73.43–87.73%, 72.51–82.78%, 74.78–87.18%, 76.34–87.50%, 79.11–85.45%, 74.86–86.47%, 82.41–84.21%, 72.46–84.69% (data shown in Table S2), respectively.

### 2.6. Characterization of SMs

The hydrodynamic diameters and Zeta potential (ZP) of SMs were carried out using a Zeta Potential/Particle Size analyzer (Brookhaven Instruments Corporation, Holtsville, NY, USA). The morphology and particle size of the SMs were measured using transmission electron microscopy (TEM) (JEM-2100, JEOL, Tokyo, Japan). The diluted SMs samples (1 mg/mL) were dropped onto a copper grid and allowed to stand for 3 min. The sample was dried with filter paper alongside staining with phosphotungstic acid solution (2%, w/v) for 1–2 min followed by air-drying. Subsequently, the prepared samples were viewed under TEM operated at an accelerating voltage of 200 kV.

### 2.7. 6-Shogaol Loading and Encapsulation Efficiency

Drug loading (DL) and encapsulation efficiency (EE) were measured as follows. Freeze-dried 6-shogaol micelle (1 mg) was dissolved in 5 mL of acetonitrile. After ultrasonication for 5 min, the solution was filtered through a 0.45-µm filter membrane. The drug concentration in the filtrate was quantified using HPLC. The EE and DL of micelles were calculated according to Equations (1) and (2), respectively.

EE (%) = Experimental drug loading/Theoretical drug loading × 100%(1)

DL (%) = Weight of drug/Weight of carrier and drug × 100%(2)

### 2.8. In Vitro Release of SMs

In vitro release experiments of 6-shogaol stock solution and SMs were measured via the dialysis method under sink conditions. Briefly, an aliquot (1.0 mL, 0.5 mg/mL) of 6-shogaol stock solution and SMs were transferred into dialysis bags (MW = 3000) and dialyzed against 80 mL of phosphate buffer solutions of different pH values (PBS, 1.2 and 7.4, respectively) at 37 °C at a constant speed of 70 rpm. Aliquots (1 mL) were taken from dialysate at predetermined times (0 h, 0.5 h, 1 h, 2 h, 4 h, 6 h, 8 h, 12 h and 24 h), and subsequently replaced with an equal volume of preheated fresh release media. The 6-shogaol concentration of collected samples filtered through a 0.22-μm cellulose nitrate membrane was analyzed with HPLC. The cumulative release (%) was calculated by the weight ratio of released 6-shogaol to total 6-shogaol.

### 2.9. Cell Viability Assay

HepG2 cells were seeded in 96-well plates at a density of 4,000 cells/well. Cells were incubated for 24 h at 37 °C under fully humidified conditions containing 5% CO_2_. The cells were then treated with a series of concentrations (5 µM, 10 µM, 20 µM, 50 µM, 75 µM, 100 µM, and 200 µM) of samples (free 6-shogaol and SMs) in 100 µL culture medium. After incubation for 72 h, 20 µL of MTT solution (5 mg/mL) was added into each well, and the plate was incubated further at 37 °C for 4 h. After the addition of DMSO (100 µL) to solubilize the formazan crystals, the optical density was determined using a microplate plate reader (BioTek, Winooski, Vermont, USA) plate reader at 595 nm. The cell viability rate (VR) was calculated using the following equation: VR (%) = (A^s^ − A^0^)/(A^1^ − A^0^) × 100%, where A^s^, A^0^ and A^1^ are the absorbance of sample group, negative and positive control group [34].

### 2.10. In Vivo Sample Treatment

Tissue homogenate samples (liver—0.3 g) were prepared after the addition of normal saline (2 mL) and homogenate for 2 min. Afterward, 50 µL of internal standard solution (curcumin, 5 µg/mL) was added to 0.5 mL of each of the homogenates or 200 µL of plasma. After mixing completely, the samples were extracted with 3 mL ethyl acetate. The upper organic layer was taken and dried with nitrogen at 40 °C after centrifugation at 4000 rpm for 10 min. The samples were later reconstituted in 0.1 mL of 65% methanol as mobile phase. The supernatant was analyzed using HPLC after centrifugation at 10,000 rpm for 10 min.

### 2.11. Oral Pharmacokinetic Study of Micelles

Twelve male Sprague-Dawley (SD) rats (male, 5–7 weeks, 220 ± 20 g) were obtained from Laboratory for Animals Care and Use of Jiangsu University, Jiangsu (Zhenjiang, Jiangsu Province, China, SCXK (SU) 20130036). This study was approved by the Experimental Animal Care and Use Committee of the Science and Technology, Zhenjiang, China. Animal are complied with institutional guidelines and regulations (JUC-201727462). All the rats were maintained in an environmentally controlled room (23 °C, 12 h dark light cycle) with free access to standard laboratory food and water for 7 days before the experiments. Before oral administration, the entire rats were randomly divided into two groups with 6 cases each, which were then fasted for 12 h but given only water. The two groups were orally administrated with free 6-shogaol suspension [5 mg/mL, in 0.5% (v/v) castor oil mixture, 100 mg/kg], and SMs of equivalent 6-shogaol dose (5 mg/mL, in normal saline, 100 mg/kg). Blood samples (600 µL each) were withdrawn at appointed time periods (0.25, 0.5, 0.75, 1, 1.5, 2, 4, 6, 8 and 12 h). Plasma (200 µL each) was obtained from blood samples after centrifuging at 3000 rpm and then were pretreated as described under the “Sample pretreatment” section. The 6-shogaol content in supernatant was determined using HPLC analysis. The pharmacokinetic parameters of peak concentration (C_max_), time of peak concentration (T_max_), area under the plasma concentration-time curve (AUC_0–12 h_) and elimination half-life (t_1/2_) were determined using BAPP 2.3 pharmacokinetic software supplied by the Center for Drug Metabolism of the China Pharmaceutical University.

### 2.12. Tissue Distribution of SMs

Thirty Kunming mice (male, 4–6 weeks, weighing 18 g–22 g) were obtained from Laboratory for Animals Care and Use of Jiangsu University, Jiangsu (Zhenjiang, Jiangsu Province, China, SCXK (SU) 20130036). Prior to oral administration, all of the mice were randomly and equally divided into 2 groups, and then fasted for 12 h and fed with only water. The first group was orally administered with free 6-shogaol suspension [5 mg/mL in 0.5 % (v/v) castor oil mixture] at a dose of 100 mg/kg, while the other group was treated with the same content of 6-shogaol in the SMs. At specific times (0.5 h, 1 h and 4 h) after the oral administration, the blood was taken from the mice’s eye socket and the tissues viz. heart, spleen, lung, liver, kidney, stomach, small intestine and brain were collected from the sacrificed mice. All tissue samples were rinsed with ice-cold saline and blotted dry. The samples were then weighed and frozen at −20 °C for further analysis. The drug content of tissue was measured via HPLC analysis. The ratio of the content of tissue and blood was calculated as the relative targeting drug delivery index (DDI).

### 2.13. Determination of Hepatoprotective Effect in Vivo

The hepatoprotective effect in vivo was tested in mice using CCl_4_-induced hepatotoxicity model. Kunming mice were randomly divided into five groups, each of ten cases. Mice of the 1^st^ (normal control) and 2^nd^ (intoxicated control) groups were treated with a single dose of gastric gavage physiological saline (20 mL/kg body weight) via oral administration. Animals of the 3^rd^, 4^th^ and 5^th^ groups were respectively given sylimarin (100 mg/kg body weight per day), free 6-shogaol (100 mg/kg) and SMs (100 mg/kg) orally each day for one week. At 6 h after last dose, the hepatotoxicity model was induced in all mice except those in the normal group by oral administration of CCl_4_ at a single dose of CCl_4_ and castor oil mixture (0.3 % v/v, 5 mL/kg body weight). At 24 h after CCl_4_ oral administration, the blood was taken though the mice’s eye socket. Then, serum was immediately collected after centrifugation of clotted blood at 3000 rpm at 4 °C for 15 min. Liver samples were collected immediately from the sacrificed mice and a portion of the left liver lobe was collected and fixed in 10% formalin for 24 h. The rest of liver was stored at −80 °C for subsequent measurement.

The activities of serum AST and ALT were measured with commercially available test kits. In brief, ALT and AST activities were analyzed by the dinitrophenyl-hydrazine method [35]. The thawed liver sample was homogenized in 0.1 g/mL of ice-cold phosphate buffer (pH 7.4). The protein content in liver samples was detected by a BCA protein assay kit. The GSH-Px, T-SOD, the levels of MDA and protein content were assayed using commercially available test kits (as indicated in the supplementary material). The liver preserved in 10% formalin solution was then embedded in paraffin for subsequent slicing and staining with hematoxylin and eosin (H&E).

### 2.14. Data Analysis

All the data are expressed as the mean ± standard deviation (SD). The statistical significance of differences between treatment groups in the pharmacokinetic study, tissue distribution and hepatoprotective effect was assessed using an unpaired student’s t-test. A *p*-value of less than 0.05 represents statistically significant differences.

## 3. Results and discussion

### 3.1. Morphology, Particle Size and Zeta Potential

**Figure 1:**
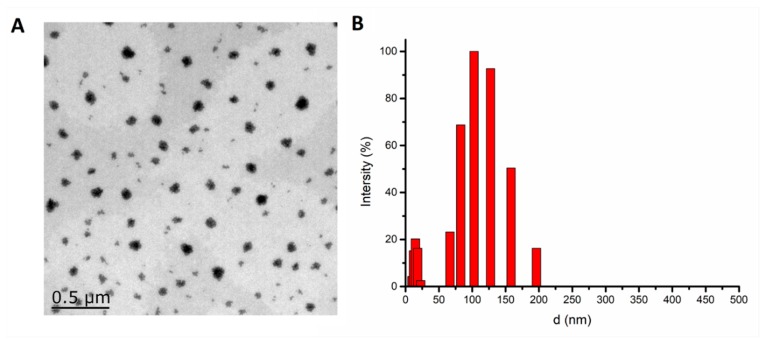
Transmission electron microscope (**A**) and the dynamic light scattering (**B**) results of 6-shogaol loaded micelles (SMs). [10/1, the mass of methoxypolyethylene glycol (MW = 2000)-linoleate acid conjugate (mPEG_2k_-LA)/6-shogaol].

mPEG2k-LA was synthesized by esterifying mPEG2k with linoleic acid (Figure S8). The critical aggregation concentration (CAC) of mPEG_2k_-LA micelle was 0.1372 mg/mL (Figure S9). The measurement of mean diameter, ZP, EE and DL were among the factors to be considered in the optimal formulation. According to the characterization results of SMs in Table 1, it was shown that the d and DL of micelles were increased by the percentage of 6-shogaol and mPEG_2k_-LA. On the contrary, the ZP and EE decreased as the percentage of mPEG_2k_-LA and 6-shogaol increased. The increase of particle size was from 27.5 nm at weight rate of 0.05/1 nm to 76.8 nm at the weight rate of 0.1/1. The remarkable increase of particle size was due to the increase in 6-shogaol encapsulation in the micellar hydrophobic interior [36]. However, the particle size increased slightly from 76.8 nm at the weight rate of 0.1/1 to 93.51 nm at 0.2/1. This phenomenon could be potentially due to the absence of 6-shogaol in the micellar core vis à vis its absorbance on the surface of micelles causing ZP to remarkable decrease as the percentage of mPEG_2k_-LA and 6-shogaol increased (ZP from −3.61 at the rate of 0.1/1 to −11.77 at the rate of 0.2/1). Therefore, the rate of 0.1/1 was selected as the optimal formulation ratio of 6-shogaol and mPEG_2k_-LA with appropriate EE (81.6%), DL (7.3%) and PDI (0.088). The morphology study using the TEM showed that the SMs were spherical in shape (Figure 1A and Figure S10) with particle sizes near to 90 nm, which is close to the results detected via DLS (Figure 1B). Interestingly, it was found that the SMs had a clear core-shell structure which was also consistent with the characterization variation of different percentage of mPEG_2k_-LA and 6-shogaol.

### 3.2. Solubility Test

The solubility test results showed that the solubility of 6-shogaol in water, pH 1.2 and 7.4 PBS was 21.39 ± 2.53 µg/mL, 26.42 ± 1.84 µg/mL and 24.76 ± 3.01 µg/mL, respectively. However, the SMs solution prepared at the concentration of 12 mg/mL was a transparent liquid. This indicated that SMs could markedly improve the solubility of 6-shogaol.

### 3.3. In Vitro Release of SMs

**Figure 2:**
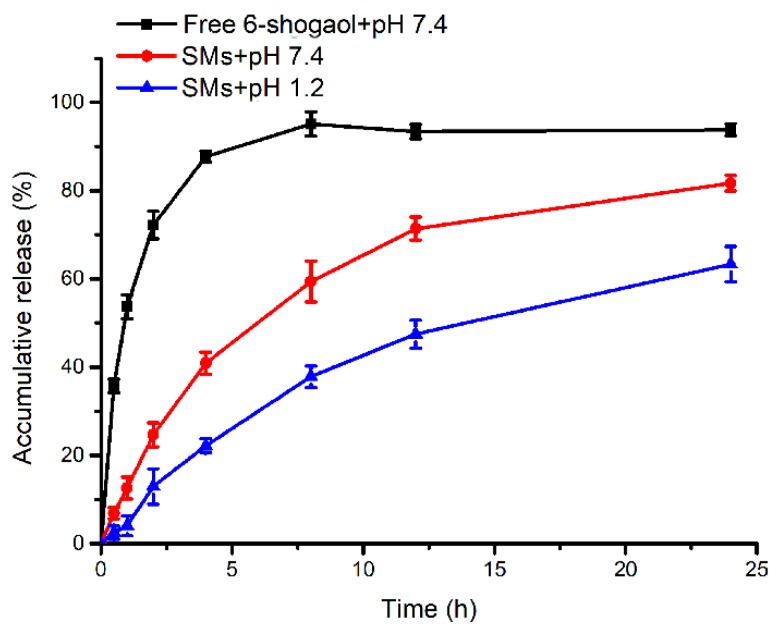
In vitro release of free 6-shogaol and SMs in different medium for 24 h.

In present study, pH 1.2 and 7.4 phosphate buffer solutions (PBS) were applied to simulate the environment of gastric juice and intestinal fluid, respectively. The in vitro release behavior of 6-shogaol from micelles was evaluated in PBS (pH 1.2 and 7.4) under sink conditions at 37 °C for 24 h. The cumulative release result of 6-shogaol from micelles is shown in Figure 2. It was observed that free 6-shogaol in DMSO could release 87 % in artificial intestinal juice during 4 h. It was indicated that 6-shogaol could completely diffuse across the dialysis bag. Over the entire period of the study, a sustained 6-shogaol release totaling approximately 81 % was accumulated from the micelles in the intestinal fluid within 24 h. Notably, the cumulative release rate of 6-shogaol from the micelles in the intestinal juice was higher than that in the artificial gastric juice. According to the results (Figure S7), the peak of mPEG_2k_-LA after incubation in PBS (pH = 7.4) at 37 °C for 24 h was longer compared with before incubation. It was observed that the mPEG_2k_-LA begun to degrade to smaller molecular compounds within 24 h, which could potentially serve as the underlying mechanism for the release of 6-shogaol from the micelles. These results indicate that 6-shogaol encapsulated in micelles may be slowly released along the degradation of mPEG_2k_-LA which was more easily hydrolyzed in artificial intestinal juice.

### 3.4. In Vitro Anti-Tumor Activity

**Figure 3:**
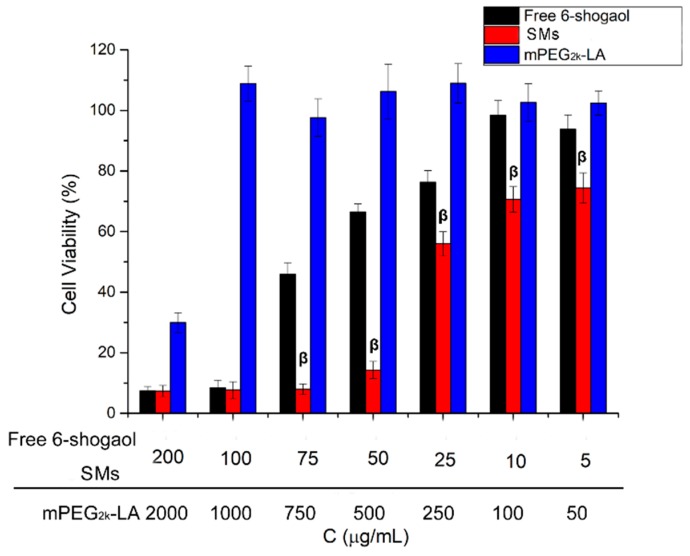
Cell viability of different samples (mPEG_2k_-LA, free 6-shogaol and SMs) with varied concentrations for 72 h using a 3-(4,5-dimethyl-2-thiazolyl)-2,5-diphenyl-2-H-tetrazolium bromide (MTT) bioassay against HepG2 cells. ^β^ *P* < 0.01 SMs versus free 6-shogaol.

The in vitro inhibitory effects of free 6-shogaol and SMs on HepG-2 cells were determined via MTT assay using mPEG_2k_-LA as controls. As show in Figure 3, it was revealed that the proliferation of HepG-2 cells could be inhibited by both free 6-shogaol and SMs in a dose-dependent manner. Astonishingly, the cell viability of SMs on HepG2 was significantly lower than that of free 6-shogaol at low concentration range (5–50 µg/mL). The micelles carrier material mPEG_2k_-LA showed almost no cytotoxicity on HepG2 cells at high concentration (1 mg/mL). Therefore, the reason for the increased cytotoxicity is attributed to its entrapment in micelles with enhanced solubility, rather than the cytotoxicity of the carrier- mPEG_2k_-LA [37]. The enhanced in vitro cancer growth inhibition of SMs was possibly due to the different cellular uptake mechanism of the micelles with free 6-shogaol [38]. The encapsulated drug in micelles entered the cell via the carrier-mediated endocytosis due to its extra small size. However, the cellular uptake of free 6-shogaol could be through passive diffusion [39]. Besides, mPEG_2k_-LA, like some carrier materials including PLGA and TPGS, may have the inhibition ability of active efflux transporters such as p-glycoprotein [27,40]. The mPEG_2k_-LA exhibited cytotoxicity at 2 mg/mL probably owing to the activity of linoleic acid [41].

The anti-tumor mechanism of 6-shogaol was found through the induction of cell cycle arrest and apoptosis in human hepatoma cells. 6-shogaol has also been reported to induce apoptosis in human hepatocellular carcinoma cells in relation to caspase activation and endoplasmic reticulum (ER) stress signaling via PERK/eIF2a pathway [42]. Meanwhile, 6-shogaol was established to reduce constitutive and interleukin (IL)-6-induced STAT3 activation while inhibiting both constitutive and TNF-a-induced NF-kB activity to induce the apoptosis of human (LNCaP, DU145, and PC3) and mouse(HMVP2) prostate cancer cells [43].

### 3.5. Oral Pharmacokinetic Study of Micelles

**Figure 4:**
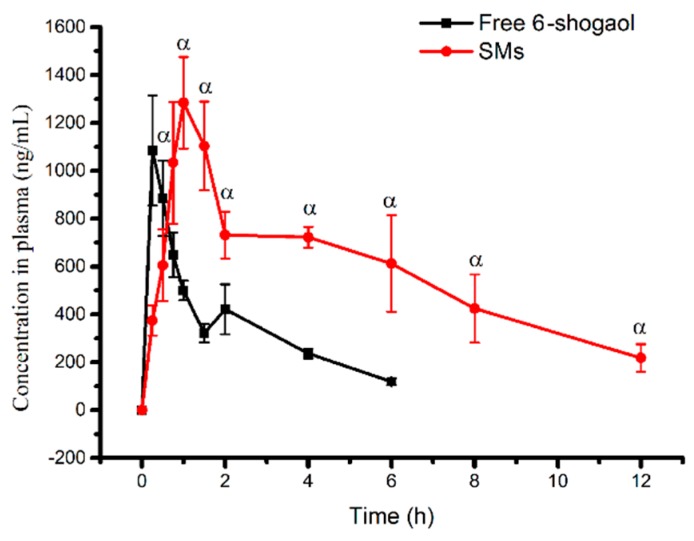
Mean plasma concentration-time profiles of 6-shogaol after oral administration of a single 100 mg/kg dose each of SMs and free 6-shogaol to rats. Results are presented as the Mean ± SD (n = 5). ^α^ *P* < 0.05, SMs versus free 6-shogaol.

As shown in the plasma concentration-time curves, the plasma 6-shogaol concentration of SMs was greater than that of free 6-shogaol after a 0.75 h time point (Figure 4). The profiles and absorption of SMs also showed significant increases in the parameters (AUC_0–12 h_, t_1/2_, MRT, T_max_, C_max_) (Table 2). Specifically, the SMs took a 2.78-fold longer time to reach the maximum plasma concentration compared with the free 6-shogaol. The C_max_ of the encapsulated 6-shogaol was also approximately 1.18-fold higher than the free drug suspension, while the AUC_0–12 h_ also showed an approximate 3.2-fold increase. Importantly, t_1/2_ and MRT were significantly prolonged after forming the micelles due to the slower release rate compared to the free 6-shogaol suspension. The enhancement of absorption and bioavailability could be ascribed to the increase in solubility and small particle size [44]. Collectively, these findings suggest that SMs could significantly improve the oral bioavailability and absorption with the potential to further enhance tissue distribution and in vivo bioactivity.

### 3.6. Tissue Distribution of SMs

**Figure 5:**
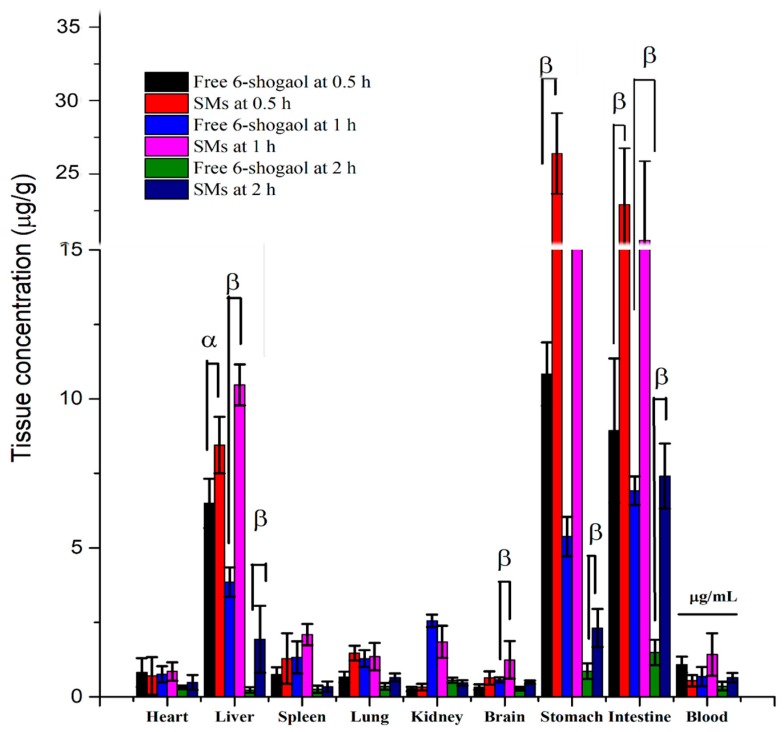
Concentration of 6-shogaol in the heart, liver, spleen, lung, kidney, stomach, small intestine brain and blood following oral administration of a single 100 mg/kg dose of free 6-shogaol and SMs to mice at 0.5 h, 1 h and 2 h point. Results are presented as the Mean ± SD (n = 5). ^α^ *P* < 0.05 SMs versus free 6-shogaol. ^β^ *P* < 0.01 SMs versus free 6-shogaol.

The tissue distribution results of free 6-shogaol and SMs after oral administration at 0.5 h, 1 h and 2 h are shown in Figure 5, respectively. It was observed that free 6-shogaol accumulated significantly in the stomach, small intestine and liver at 0.5 h and 1 h due to the liver first pass effect. However, the liver distribution of free 6-shogaol at 2 h declined sharply and was lower than other tissues such as heart, spleen, lung, kidney and brain. Notably, the 6-shogaol was mostly distributed in the kidney with the exception of the stomach at 2 h. This indicated that free 6-shogaol would be rapidly catabolized in the liver and excreted in the kidney. The tissue distribution results of free 6-shogaol was consistent with other reports [45]. Compared to free 6-shogaol, the tissue distribution of SMs in the liver, lung and stomach was significantly enhanced due to the ability of the micelle to improve the solubility and oral absorption of the lipophilic drug. Furthermore, the relative liver delivery index (DDI) of SMs at 2 h was significantly higher than that of the free 6-shogaol (relative to blood, 1.45 vs 0.69, c/c). In contrast, the relative kidney distribution of SMs at 2 h was significantly lower than that of free 6-shogaol (relative to blood, 0.73 vs 1.53, c/c). More significantly, the distribution in brain of 6-shogaol was enhanced after its encapsulating in the SMs. This is because smaller micelles (50 nm–100 nm) could enter the lymphatic system in the intestine and delivery 6-shogaol into the brain and liver [22]. These results demonstrated that SMs could improve the accumulation of 6-shogaol in tissue especially the brain and liver tissue.

### 3.7. Hepatoprotective Effect in Vivo

**Figure 6:**
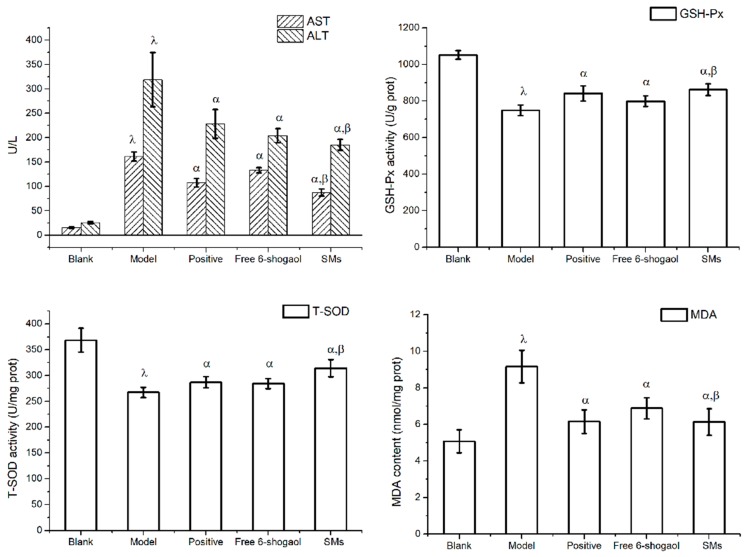
Effects of free 6-shogaol and SMs on serum ALT, AST activity (**A**) and liver GSH-Px (**B**), T-SOD (**C**) and MDA (**D**) activity. Animals were given orally: castor oil (model group); silymarin (positive group, 100 mg/kg); free 6-shogaol (100 mg/kg); or SMs (100 mg/kg) once daily for one week prior to the administration of CCl_4_ (0.2%, i.p). Except blank group, which accepted the administration of castor oil. ^α^ *P* < 0.05 versus model group. ^β^ *P* < 0.01 versus free 6-shogaol. ^λ^ *P* < 0.05 model group versus blank group.

The results of the hepatoprotective effect of 6-shogaol and SMs on the serum ALT and AST activities are shown in Figure 6A. In the CCl_4_ intoxicated group, serum ALT and AST values were 318.5 U/L and 161.2 U/L, respectively, whereas the values of normal group were only 25.49 U/L and 15.46 U/L, respectively. The significant increase in the activities of serum ALT and AST indicated that the CCl_4_ induced liver injury model was successfully established. Oral administration of positive drug sylimarin, 6-shogaol and SMs for one week significantly reduced the activities of serum ALT by 28.4%, 36.0% and 41.78%, and as well as the activities of serum AST by 33.3%, 28.0% and 45.9%, respectively, as compared to the model group (*P* < 0.05). More importantly, the activities of serum ALT and AST in the SMs group was significantly reduced compared to the 6-shogaol group (*P* < 0.05).

The activities of GSH-Px, T-SOD and MDA in the liver were also evaluated. Significantly lowered activities of GSH-Px and T-SOD, coupled with increased MDA content were observed in CCl_4_ model group mice liver as compared with the control group (Figure 6B–D). The positive drug sylimarin, 6-shogaol and SMs also showed a significant increase in the activities of GSH-Px and T-SOD as well as a decrease in the content of MDA in the liver compared to the model group (*P* < 0.05). Notably, the SMs showed higher activities of GSH-Px and T-SOD as well as a lower content of MDA in the liver than the 6-shogaol group, even more than the positive cohort (*P* < 0.05).

**Figure 7:**
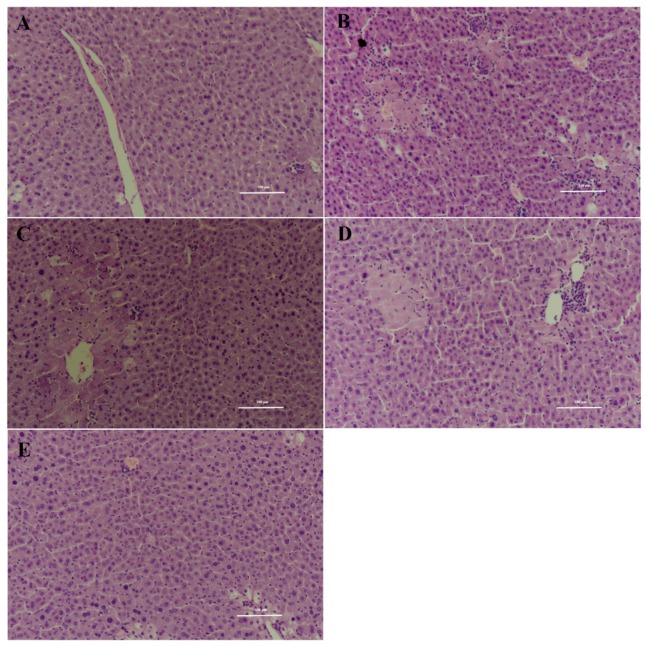
Effects of free 6-shogaol and SMs on hepatic morphological analysis (×200 H&E): Blank group (**A**), CCl_4_-model group (**B**), Positive drug (100 mg/kg) and CCl_4_ group (**C**), Free 6-shogaol (100 mg/kg) and CCl_4_ group (**D**), SMs (100 mg/kg) and CCl_4_ group (**E**). The scale bar: 100 μm.

The hepatoprotective effects of 6-shogaol and SMs were further confirmed using conventional histological assessment (Figure 7). The histology of the liver sections of the control group showed normal hepatic cells with a well-preserved cytoplasm and a prominent nucleus and nucleolus (Figure 7A). The stained sections of CCl_4_ model group revealed extensive liver injuries characterized by obvious soma shrinking, dissociation of the hepatic cord, large area hepatic necrosis, inflammatory cell infiltration and ballooning degeneration (Figure 7B). CCl_4_-intoxicated mice pretreated with 6-shogaol and positive group showed moderate hypertrophy of hepatocytes with a relatively intact central vein, diminished hepatic necrosis and reduced inflammatory cell (Figure 7C). However, CCl_4_-intoxicated mice pretreated with SMs only exhibited individual cell ballooning degeneration compared to CCl_4_ model group. These results indicated that 6-shogaol has certain hepatoprotective effects against the CCl_4_-induced liver injury through restoration and maintenance of the activities of T-SOD and GSH-Px in CCl_4_-damaged liver. The underlying mechanism of hepatoprotective effects of 6-shogaol might be attributed to anti-inflammatory and antioxidative activities of its phenolic structure [46,47]. More significantly, SMs might improve the hepatoprotective effects of 6-shogaol due to enhanced oral bioavailability coupled with liver accumulation.

## 4. Conclusions

Novel mPEG_2k_-LA micelles were successfully developed for oral delivery of the hydrophobic compound 6-shogaol. A high drug encapsulation of 80% was achieved under drug loading capacity of 7%, which greatly enhanced the 6-shogaol delivery efficiency versus general oral delivery systems. The developed micelles (SMs) showed a slower release rate than the free 6-shogaol. Moreover, SMs significantly improved the anti-cancer activity of 6-shogaol *in vitro*. In addition, SMs showed enhanced oral bioavailability and liver distribution compared to free 6-shogaol. The in vivo liver protection study also demonstrated that SMs markedly reduced the activities of serum AST, ALT and liver MDA levels, while remarkably increased the antioxidant activities (GSH-Px, T-SOD). Therefore, the novel micelle is expected to serve as a promising carrier for 6-shogaol to enhance its cancer treatment and hepatoprotection.

## Figures and Tables

**Table 1:** Physicochemical characterization of the micelles made of mixtures of 6-shogaol and methoxypolyethylene glycol (MW=2000)-linoleate acid conjugate (mPEG_2k_-LA) (mean values ± standard deviation, n = 3): measurement of mean diameter (D), Zeta potential (ZP) and polydispersity index (PDI), entrapment efficiency (EE) and drug loading (DL).

Mass Ratio	Ratio	ZP (mV)	D (nm)	PDI	EE%	DL%
mPEG_2k_-LA/6-shogaol	20:1	−2.05	27.54	0.105	98.4	4.7
	10:1	−3.61	76.8	0.088	81.6	7.3
	5:1	−11.77	93.51	0.120	68.9	12.4
	2:1	−12.16	104.17	0.150	35.4	15.0
	1:1	−17.95	112.29	0.189	22.9	17.5

**Table 2:** Pharmacokinetic parameters of free 6-shogaol and 6-shogaol loaded micelles (SMs) after oral administration in rats (mean ± SD).

Parameters	Free Drug	SMs
C_max_ (ng∙mL^−1^)	1085.3 ± 230.1	1284.3 ± 191 ^α^
T_max_ (h)	0.33 ± 0.14	0.92 ± 0.14 ^β^
t_1/2_ (h)	2.05 ± 0.09	4.65 ± 0.39 ^β^
MRT (h)	2.06 ± 0.05	7.48 ± 2.33 ^β^
AUC_0–4 h_ (h∙ng∙mL^−1^)	2123.2 ± 193.6	6834.1 ± 826.1 ^β^

^α^*P* < 0.05, compared with free 6-shogaol. ^β^ *P* < 0.01, compared with free6-shogaol.

## Supplementary Materials

The following are available online at https://www.mdpi.com/1999-4923/11/3/107/s1, Figure S1: The ESI-MS of 6-shogaol, Figure S2: The ^1^H-NMR of 6-shogaol (CDCl3, ppm), Figure S3: The chromatogram of purified 6-shogaol and the structure of 6-shogaol (insert), Figure S4: HPLC chromatograms of blank micelles (A), 6-shogaol (B) and sample of 6-shogaol-loaded micelles (C), Figure S5: Chromatograms of 6-shogaol and internal standard in rat plasma, Figure S6: Chromatograms of 6-shogaol and internal standard in mouse heart, liver, spleen, lung, kidney, brain, stomach, intestine homogenate and plasma. Figure S7: The gel chromatogram of mPEG_2k_-LA and mPEG_2k_-LA after incubation at PBS = 7.4 for 24 h, Figure S8: Synthesis route of mPEG2k-LA, Figure S9: The critical aggregation concentration (CAC) of mPEG_2k_-LA micelles was measured by Fluorescence probes (pyrene), Figure S10: The TEM micrograph of 6-shogaol loaded micelles (6-shgoaol/mPEG_2k_-LA = 1/10). Table S1: Linear ranges, standard curves and regression coefficients of 6-shogaol in different biosamples. Table S2: The precision (intra-day and inter-day precision), accuracy (relative recovery) and extract recovery of analysis method in vivo.

## Abbreviations

SMs, 6-shogaol loaded in micelles;
DL, Drug loading;
ZP, zeta potential;
mPEG_2K_-LA, methoxypolyethylene glycol (MW = 2000)-linoleate acid conjugate linoleate acid conjugate;
FBS, fetal bovine serum;
PBS, phosphate buffer saline;
HPLC, high performance liquid chromatography;
PDI, polydispersity index;
TEM, transmission electron microscopy;
ESI-MS, Electrospray Ionization Mass Spectrometry;
NMR, nuclear magnetic resonance spectroscopy;
DMSO, dimethylsulfoxide;
rpm, revolutions per minute;
MRT, mean residence time
