# Supplementary material for: Enhanced Oral Bioavailability, Anti-Tumor Activity and Hepatoprotective Effect of 6-Shogaol Loaded in a Type of Novel Micelles of Polyethylene Glycol and Linoleic Acid Conjugate
Source: Pharmaceutics. 2019 Mar 6;11(3):107. doi: 10.3390/pharmaceutics11030107 (PMC6470752; doi:10.3390/pharmaceutics11030107)
Supplement: Supplementary file 1 [file pharmaceutics-11-00107-s001.pdf]

pharmaceutics

Supplementary

Enhanced Oral Bioavailability, Anti-Tumor Activity and Hepatoprotective Effect of 6-Shogaol Loaded in a Type of Novel Micelles of Polyethylene Glycol and Linoleic Acid Conjugate

Huiyun Zhang †, Qilong Wang †, Congyong Sun †, Yuan Zhu, Qiuxuan Yang, Qiuyu Wei, Jiaxin Chen, Wenwen Deng, Michael Adu-Frimpong[a], Jiangnan Yu * and Ximing Xu *

Center for Nano Drug/Gene Delivery and Tissue Engineering, Department of Pharmaceutics, School of Pharmacy, Jiangsu University, Zhenjiang 212013, China

* Correspondence: xmxu@ujs.edu.cn (X.X.); Tel:+86-5118-503-845; Fax:+86-5118-503-8451; yjn@ujs.edu.cn (J.Y.); Tel:+86-5118-503-845; fax:+86-51185-038-451

† These authors contributed equally to this work.

Received: 19 December 2018; Accepted: 1 March 2019; Published: date

Methods

1. ESI-MS and ^{1}H-NMR

The ESI-MS data was acquired in a positive ion mode using a Thermo Finnigan LXQ Dec XP and Ion Trap Mass Spectrometer instrument from Thermo Finningan (San Jose, CA). The instrument was equipped with an electrospray ion source (ESI) and an Xcalibur® system manager data acquisition software. Sample solutions (2 μL, 5 mg/mL) were injected into the ESI source using a syringe pump with a mass scan range of 100 m/z —1000 m/z. The conditions for the ion trap mass spectrometer were as follows: spray voltage, 4.5 kV; source current, 80 μA; capillary temperature, 325 °C; capillary voltage, 30 V; tube lens offset, 120 V; multi-pole 1 offset, 6 V; multi-pole 2 offset, 10 V; and sheath gas flow (N2), 35 A.U.

Proton nuclear magnetic resonance (^{1}H-NMR) spectra were run at 400 MHz on a NMR spectrometer (Bruker AVANCE Ⅱ, Switzerland) and the chemical shifts were in ppm down field from $CDCl_3$.

2. Synthesis of $mPEG_{2k}$-LA

Linoleic acid (0.5g, 1.78 mmol), $mPEG_{2k}$ (1g, 0.5 mmol), DCC (0.4g, 1.94 mmol) and DMAP (0.24 g, 1.96 mmol) were added to dichloromethane (6 mL) at room temperature. The solution was stirred for 2 h under nitrogen atmosphere, filtered to remove DCU, washed with saturated sodium chloride solution, dried over Na_2SO_4 and concentrated in vacuo. The residue was washed with petroleum ether thrice and dried under vacuum to give mPEG2k-linoleic acid (mPEG2k-LA) as a white solid (1.09g, 62 %).

3. Assay validation of 6-shogaol measured in tissue and plasma

3.1. System suitability.

System suitability was performed using the blood plasma and tissue as the working biological samples. Blank plasma and tissue, plasma and tissue containing 6-shogaol and Curcumin were treated as indicated in section 2.10. The system suitability was evaluated by determination of 6-shogaol by HPLC method.

3.2. Linearity.

Based on the results of the HPLC, the linearity was calculated by regression analysis. After plotting the peak area ratios of 6-shogaol to the internal standard (Curcumin) as ordinate (y) and the concentration of 6-shogaol as abscissa (x), the calibration curves, linear ranges and regression coefficients of plasma and tissues were analyzed.

3.3. Intra-day and Inter-day precision.

QC samples (in vivo) were prepared as described in section 2.10. Aliquots (20 μL) of QC samples were injected into HPLC and detected five consecutive times within a day. The ratios (f) of the peak areas of 6-shogaol to the internal standard were calculated. Intra-day RSD was calculated by the substitution of the ratios (f) into the standard curve. Similarly, each QC sample (20 μL) was injected into HPLC to detect 6-shogaol content in five successive days' determination, and the inter-day RSD was calculated.

3.4. Accuracy.

The accuracy was estimated by method recovery. Aliquot of QC samples (20 μL) was injected into the HPLC to detect the content of 6-shogaol. The ratios (f) of the peak areas of 6-shogaol to IS were calculated, and then substituted into the calibration curve to calculate the contents of 6-shogaol. The method recovery was calculated according to Eqn. (3.1).

$$\text{Method Recovery}(\%) = \frac{\text{Measured amount}}{\text{Theoretical amount}} \times 100\% \qquad 3.1$$

3.5. Extraction recovery.

QC samples (20 μL) were injected into HPLC. The peak areas of 6-shogaol in QC samples were obtained. Aliquot (20 μL) of the mixed aliquots of 6-shogaol and Curcumin with QC concentration were injected into HPLC and identified accordingly, and the peak areas of 6-shogaol in mixed aliquots were calculated. The extraction recovery was calculated according to Eqn. (3.2)

$$\text{Absolute Recovery } (\%) = \frac{\text{Area}}{\text{Area}'} \times 100\% \qquad 3.2$$

Where, Area was the peak area of 6-shogaol through overall extraction procedure and Area′ was the peak area of 6-shogaol through direct dilution.

The absolute recoveries of analytes were determined by comparing the peak areas of samples (three concentrations in five replicates) through overall extraction procedure with that of samples diluted by methanol directly.

The absolute recoveries of analytes were determined by comparing the peak areas of samples (three concentrations in five replicates) through overall extraction procedure with that of samples diluted by methanol directly.

4. The critical aggregation concentration (CAC) of mPEG-LA micelles

The CAC of mPEG-LA micelles was determined using fluorescence probe pyrene as reported earlier [1]. A series of samples of designed concentration of mPEG-LA solution (Figure S10) with trace pyrene were determined by RF-5301 PC spectrofluorophotometer (Shimadzu, Japan). Each solution was excited at 334 nm, and fluorescence spectra were recorded between 300 nm and 500 nm. The excitation slid width was set at 10 nm and the emission slit width was 3 nm. Intensity of peak 375 nm (I375) and peak 384 nm (I384) were obtained and all the fluorescence spectra were collected (Figure S10).

5. Glutathione Peroxidase Assay.

Glutathione peroxidase (GSH-Px) activity was expressed as nanomoles of NADPH oxidized to NADP per minute per milligram of protein, with a molar extinction coefficient for NADPH at 340 nm of 6.22×10^6. Liver GSH-Px was assayed in a 1-mL crystal cuvette containing 0.8 mL of 100 mM potassium phosphate buffer (pH 7.0), 1 mM EDTA, 1 mM NaN_3, 0.2 mM NADPH, 1 U/mL GSH Rd, and 1 mM GSH. Five microliters of the liver homogenate and buffer was added to make a total volume of 0.9 mL. The reaction was initiated by the addition of 100 μL of 2.5 mM H_2O_2, and the conversion of NADPH to NADP was monitored with a spectrophotometer at 340 nm for 3 min. The specific enzyme activity of GSH-Px was expressed as U/g protein.

6. Determination of Superoxide Dismutase Activity.

A portion of the liver tissue was homogenized (10%, w/v) in a solution (0.32 M sucrose, 1 mM EDTA, and 10 mM Tris-HCl, pH 7.4) using a polytron homogenizer, after which it was centrifuged at 10 000 rpm (Beckman, Microfuge) at 4 °C for 30 min. The supernatant was assayed for superoxide dismutase (SOD) activity by following the inhibition of nitroblue tetrazolium (NBT) reduction. SOD activity was assayed by a reaction mixture containing 985 μL of 100 mM phosphate buffer (pH 7.4), 0.3 mM K_2H_2-EDTA, 0.5 mM NBT, and 0.1 mM xanthine. The mixture was preincubated for 3 min at 25 °C, and 10 μL of 0.02 U/mL xanthine oxidase was added to generate superoxide and induce NBT reduction. Changes in absorbance at 560 nm were recorded at 20-s intervals for 5 min. SOD activity was determined from a standard curve of the percentage inhibition of NBT reduction with standard SOD activity. Data are expressed as SOD units/mg protein as compared with the standard.

7. Determination of malonaldehyde.

The content of malonaldehyde (MDA) formation in the liver homogenate was determined using the thiobarbituric acid (TBA) method. Briefly, 250 μL of liver homogenate (10 %, w/v) was first mixed with the same volume of 50 mM potassium phosphate buffer. Then 20 μL of an ice-cold trichloroacetic acid (TCA) solution (4 % [w/v] in 0.3 N HCl) and 200 μL of TBA-reactive substance reagent (0.5% [w/v] TBA in 50 % [v/v] acetic acid) were also added. Samples were boiled and cooled, extracted with n-1-butanol, and centrifuged in a microcentrifuge for 10 min at 10 000 rpm. The butanol layer containing the TBA-reactive substances (TBARS) was read at 532 nm. The results were expressed as nmol/mg protein.

8. The degradation of mPEG-LA

The degradation of $mPEG_{2k}$-LA was measured using gel filtration chromatography. The detection method used was high-performance liquid chromatography-evaporative light scattering detection (HPLC-ELSD). The chromatographic conditions were as follows: TSK-GEL4000 column (7.5×300 mm) and column temperature of 30 °C; and a flow rate of 0.6 mL/min. The mobile phase was 0.2 % acetic acid aqueous.

The file includes

1 Figure S1-10 and Table S1-2

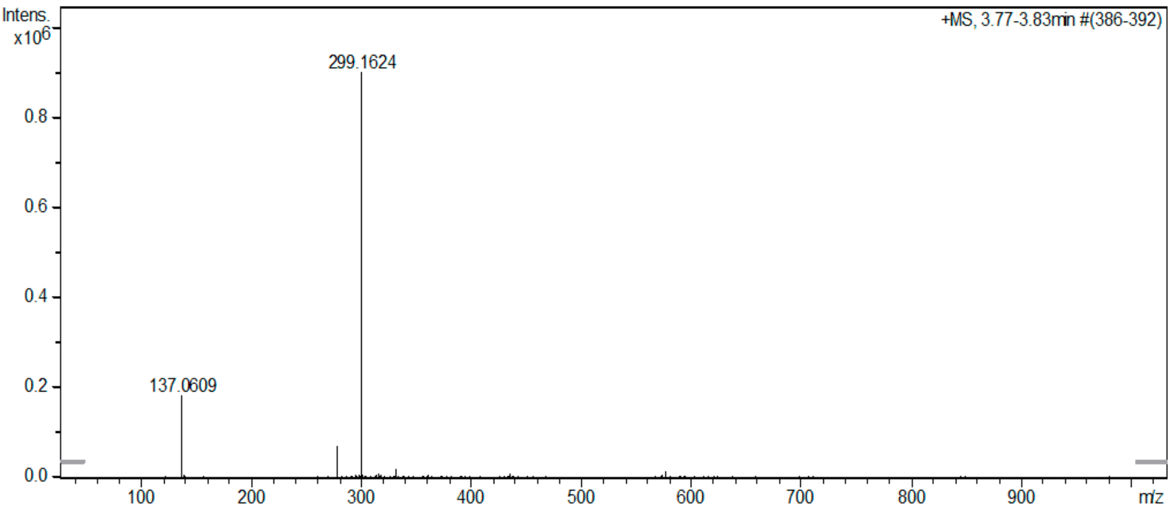

Figure S1. The ESI-MS of 6-shogaol.

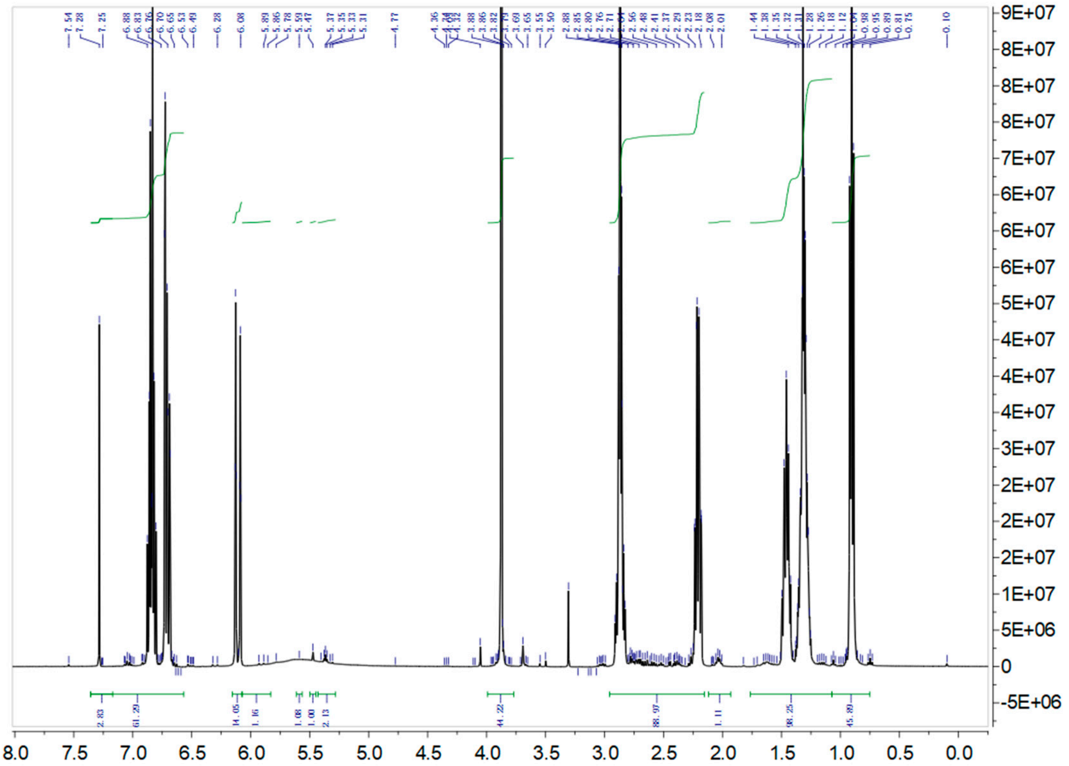

Figure S2. The ^{1}H-NMR of 6-shogaol ($CDCl_3$, ppm).

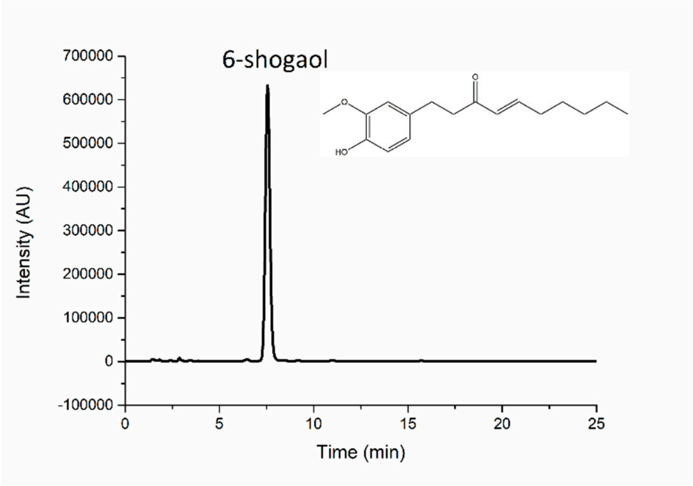

Figure S3. The chromatogram of purified 6-shogaol and the structure of 6-shogaol (insert).

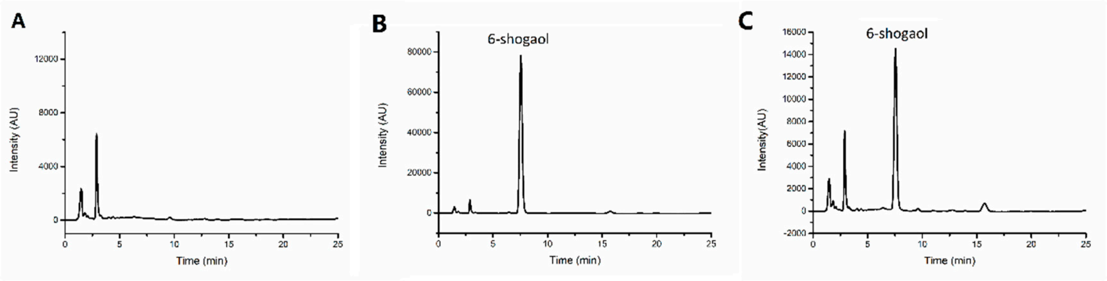

Figure S4. HPLC chromatograms of blank micelles (A), 6-shogaol (B) and sample of 6-shogaol-loaded micelles (C).

The chromatograms of the system suitability test demonstrated that 6-shogaol could achieve baseline separation, and the determinations were not interfered by excipients under the chromatographic conditions (Figure S4). The retention time of 6-shogaol was 7.55 min. The standard curve of 6-shogaol was constructed by analyzing reference solutions with different concentrations (1, 2, 5, 10, 20, 50 and 100 μg/mL). The linear regression equation of 6-shogaol was Y = 81883X-44299 (n = 3, R^2 = 0.9997), where Y represented the peak area of samples and X represented the sample concentrations of 6-shogaol. The correlation coefficient showed that the assay method was linear and acceptable for quantitative analysis. In addition, the calibration curves demonstrated good linearity over the concentration ranges of 1–100 μg/mL.

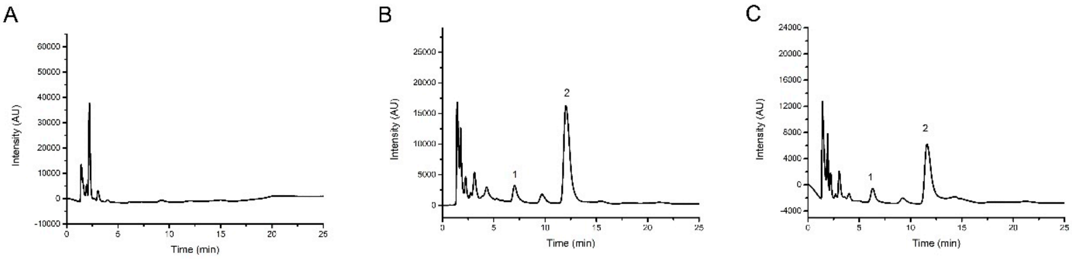

Figure S5. Chromatograms of 6-shogaol and internal standard in rat plasma: A: blank plasma; B: blank plasma spiked with 6-shogaol and internal standard; C: plasma sample after oral administration (1, internal standard; 2, 6-shogaol).

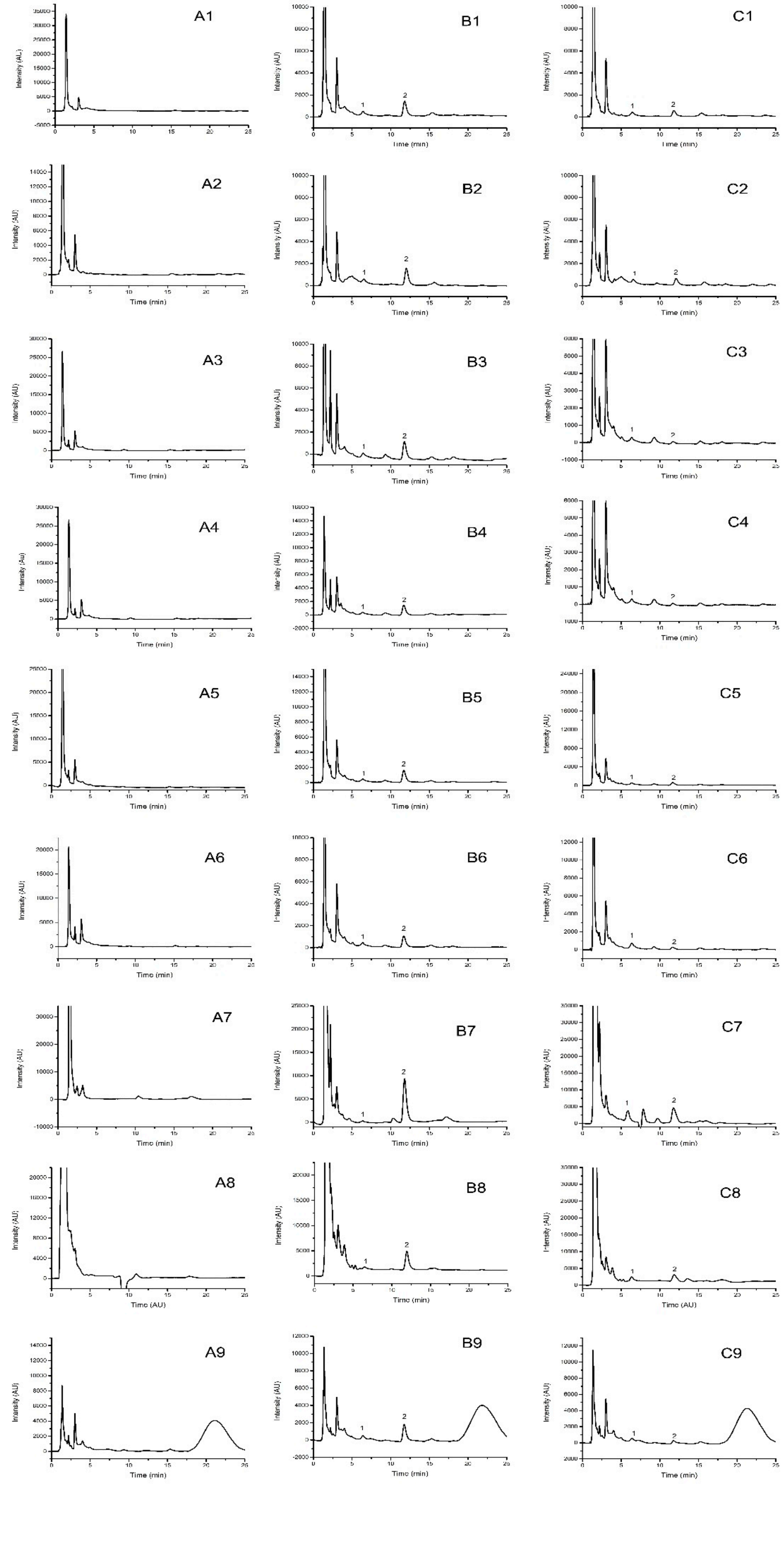
A1
B1
C1
A2
B2
C2
A3
B3
C3
A4
B4
C4
A5
B5
C5
A6
B6
C6
A7
B7
C7
A8
B8
C8
A9
B9
C9
Intensity (AU)
Time (min)
Time (AU)
1
2

Figure S6. Chromatograms of 6-shogaol and internal standard in mouse heart, liver, spleen, lung, kidney, brain, stomach, intestine homogenate and plasma (A1)~(A9): blank heart, liver, spleen, lung, kidney, brain， stomach, intestine homogenate and plasma; (B1)~(B9): blank heart, liver, spleen, lung, kidney, brain, stomach, intestine homogenate and plasma spiked with 6-shogaol and internal standard; (C1)~(C9): heart, liver, spleen, lung, kidney, brain, stomach, intestine homogenate and plasma sample after oral administration (1, internal standard; 2, 6-shogaol).

The typical equation of the standard curves of the peak area ratio to the concentration, the linear range and regression coefficients of 6-shogaol in plasma and tissue homogenates were summarized in Table S1. Standard curves were linear over the ranges 0.125 μg/mL–10.0 μg/mL in plasma, 0.05 – 7.5 μg/mL in tissue (heart, spleen, lung and brain) homogenates, 0.05 – 15.0 μg/mL in liver homogenates, 0.05 – 3.75 μg/mL in kidney homogenates and 0.15 μg/mL–5.0 μg/mL in stomach and intestine homogenates. In addition, an excellent correlation between analyzed peak area ratio and concentration of 6-shogaol was observed with $R^2 \geq 0.99$.

Table S1. Linear ranges, standard curves and regression coefficients of 6-shogaol in different biosamples (n = 3).

**Biosamples**	**Linear range (μg /mL).**	**Calibration curves**	**R^2**
Plasma	0.125 – 10.0	Y = 2.9253X – 0.7809	0.9975
Heart	0.05 -7.5	Y = 3.1008X – 0.8205	0.9948
Liver	0.05 – 15.0	Y = 2.9737X – 0.1086	0.9970
Spleen	0.05 – 7.5	Y = 2.6535X – 0.2658	0.9962
Lung	0.05 – 7.5	Y = 3.2612X – 0.2867	0.9968
Kidney	0.05 – 3.75	Y = 3.1464X – 0.2712	0.9944
Brain	0.05 – 7.5	Y = 2.7369X + 0.2063	0.9988
Stomach	0.15 – 5.0	Y = 2.9587X – 0.1682	0.9983
intestines	0.15 – 5.0	Y = 2.9368X – 0.2106	0.9963

Table S2. The precision (intra-day and inter-day precision), accuracy (relative recovery) and extract recovery of analysis method *in vivo.*

Biosamples	Nominal concentration (μg /mL)	Intra-day precision		Inter-day precision		Relative recovery (%)	Extract recovery (%)
		Measured concentration (μg /mL)	RSD (%)	Measured concentration (μg /mL)	RSD (%)		
Plasma	0.35	0.341 ± 0.01	1.27	0.362 ± 0.02	2.52	99.43 ± 1.69	86.82 ± 1.83
	1.50	1.54 ± 0.51	2.61	1.469 ± 0.62	2.42	101.52 ± 2.67	87.12 ± 2.02
	7.50	7.38 ± 0.93	2.32	7.72 ± 1.93	2.63	98.62 ± 3.31	76.23 ± 1.73
Heart	0.150	0.147 ±0.024	3.21	0.152 ± 0.002	3.62	99.52 ± 3.48	87.73 ± 4.43
	0.375	0.373 ± 0.021	2.43	0.384 ± 0.017	2.54	101.74 ± 2.58	73.43 ± 2.35
	0.750	0.754 ± 0.047	2.85	0.758 ± 0.024	3.84	101.93 ± 2.89	85.79 ± 3.53

Liver	0.150	0.151 ± 0.013	3.69	0.148 ± 0.017	3.68	107.53 ± 3.78	80.74 ± 3.68
	0.375	0.368 ± 0.024	4.18	0.382 ± 0.014	3.05	102.75 ± 2.84	82.78 ± 2.3564
	0.750	0.724 ± 0.125	3.47	0.729 ± 0.163	3.63	97.23 ± 3.82	72.51 ± 2.53
Spleen	0.150	0.157 ± 0.008	2.19	0.146 ± 0.007	1.96	104.34 ± 3.05	85.69 ± 3.48
	0.375	0.371 ± 0.028	2.37	0.369 ± 0.018	1.98	104.45 ± 3.848	74.78 ± 3.52
	0.75	0.751 ± 0.073	2.84	0.745 ± 0.015	1.68	101.80 ± 2.12	87.18 ± 2.86
Lung	0.150	0.156 ± 0.012	2.96	0.143 ± 0.007	3.66	103.41 ± 3.87	76.34 ± 3.83
	0.375	0.384 ± 0.042	2.68	0.374 ± 0.113	3.84	102.37 ± 2.32	83.37 ± 1.84
	0.750	0.776 ± 0.013	2.45	0.745 ± 0.123	3.84	98.99 ± 3.46	87.50 ± 3.45
Kideny	0.150	0.153 ± 0.004	2.84	0.147 ± 0.021	1.78	101.58 ± 2.12	85.45 ± 1.33
	0.375	0.378 ± 0.011	3.27	0.384 ± 0.009	1.74	102.11 ± 2.83	84.19 ± 2.64
	0.75	0.783 ± 0.057	3.79	0. 731 ± 0.034	1.86	97.24 ± 2.42	79.11 ± 3.43
Brain	0.150	0.152 ± 0.008	4.21	0.146 ± 0.008	2.73	97.34 ± 3.48	85.29 ± 3.31
	0.375	0.386 ± 0.017	2.84	0.370 ± 0.036	3.73	103.75 ± 3.39	74.86 ± 1.48
	0.750	0.747 ± 0.003	2.51	0.730 ± 0.076	4.34	97.29 ± 3.72	86.47 ± 3.12
Stomach	0.150	0.156 ± 0.013	2.82	0.161 ± 0.024	3.72	101.46 ± 2.52	84.42 ± 2.79
	0.375	0.371 ± 0.015	3.31	0.383 ± 0.019	3.44	102.21 ± 2.67	82.41 ± 2.59
	0.75	0.743 ± 0.048	3.57	0. 748 ± 0.024	1.97	98.24 ± 2.44	84.21 ± 3.24
Intestine	0.150	0.160 ± 0.022	3.22	0.156 ± 0.018	2.65	101.75 ± 2.84	72.36 ± 2.97
	0.375	0.365 ± 0.035	3.93	0.363 ± 0.039	3.25	98.21 ± 3.32	84.69 ± 3.14

0.75	0.752 ± 0.044	3.37	0. 758 ± 0.031	2.52	101.52 ± 2.72	83.74 ± 3.32

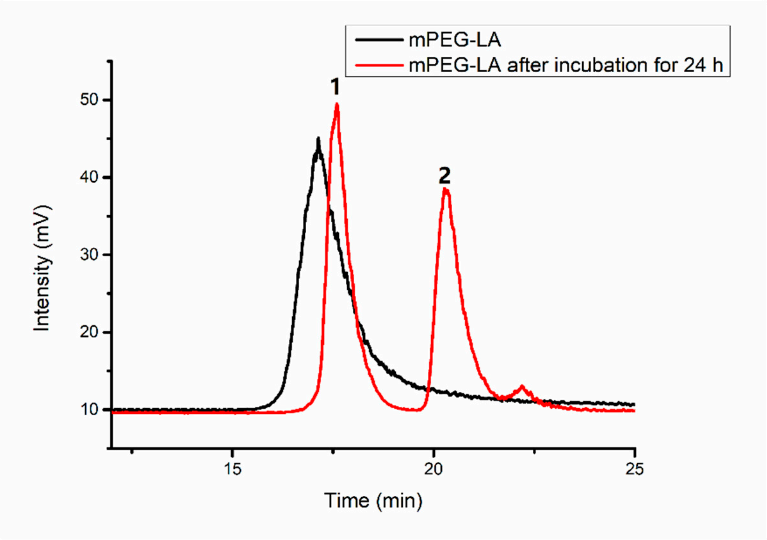

Figure S7. The gel chromatogram of mPEG-LA and mPEG-LA after incubation at PBS=7.4 for 24 h. The detection method was HPLC-ELSD. Peak 1, $mPEG_{2k}$; Peak 2, linoleic acid.

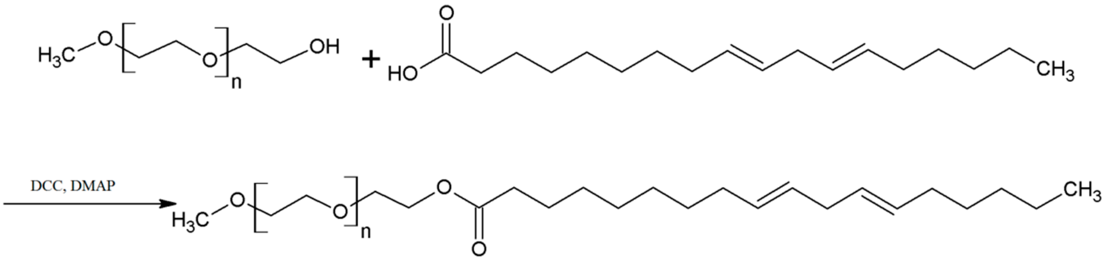

Figure S8. Synthesis route of $mPEG_{2000}$-LA.

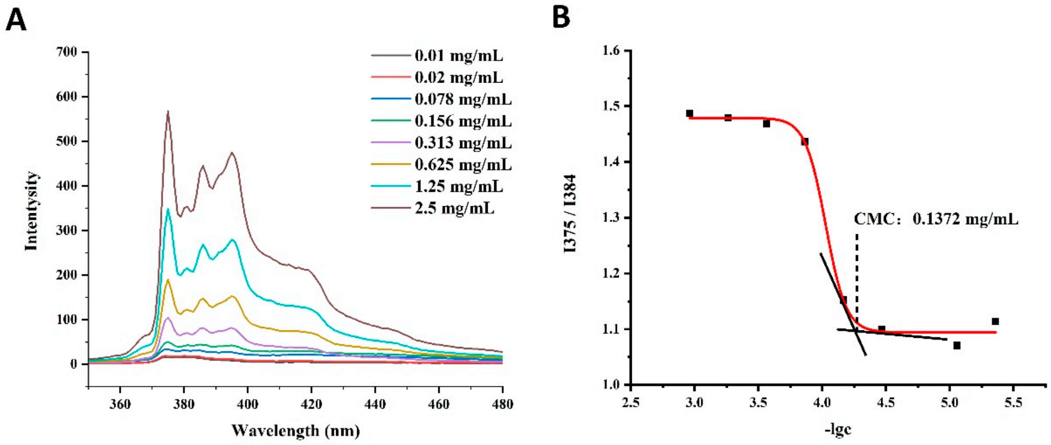

Figure S9. The critical aggregation concentration (CAC) of mPEG-LA micelles was measured by Fluorescence probes (pyrene). A: Full wavelength spectra (300 nm–500 nm) at different concentration of mPEG-LA. B: Intensity of peak 373 nm (I375) and peak 384 nm (I384).

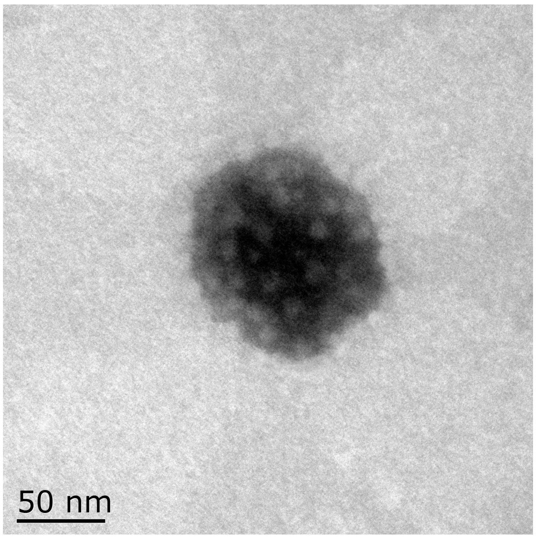

Figure S10. The TEM micrograph of 6-shogaol loaded micelles (6-shgoaol/mPEG-LA =1/10).

Reference

1 Basu, R G.; Chakraborty, I.; Moulik, S P. Pyrene absorption can be a convenient method for probing critical micellar concentration (CMC) and indexing micellar polarity. *J. Colloid Inter. Sci.* **2006**, *294* 248–254.

© 2019 by the authors. Submitted for possible open access publication under the terms and conditions of the Creative Commons Attribution (CC BY) license (http://creativecommons.org/licenses/by/4.0/).
